# Primary outcome reporting in adolescent depression clinical trials needs standardization

**DOI:** 10.1186/s12874-020-01019-6

**Published:** 2020-05-25

**Authors:** Andrea Monsour, Emma J. Mew, Sagar Patel, Alyssandra Chee-a-tow, Leena Saeed, Lucia Santos, Darren B. Courtney, Priya N. Watson, Suneeta Monga, Peter Szatmari, Martin Offringa, Nancy J. Butcher

**Affiliations:** 1 Child Health Evaluative Sciences, The Hospital for Sick Children Research Institute, 686 Bay Street, Room 11.9712, Toronto, Ontario M5G 0A4 Canada; 2 Centre for Addiction and Mental Health, Toronto, Ontario Canada; 3 Department of Psychiatry, University of Toronto, Toronto, Ontario Canada; 4 Department of Psychiatry, The Hospital for Sick Children, Toronto, Canada; 5 Institute of Health Policy, Management and Evaluation, University of Toronto, Toronto, Ontario Canada; 6 Division of Neonatology, The Hospital for Sick Children, University of Toronto, Toronto, Ontario Canada

**Keywords:** Major depressive disorder, Adolescent, Randomized clinical trial, Outcome reporting, Primary outcomes

## Abstract

**Background:**

Evidence-based health care is informed by results of randomized clinical trials (RCTs) and their syntheses in meta-analyses. When the trial outcomes measured are not clearly described in trial publications, knowledge synthesis, translation, and decision-making may be impeded. While heterogeneity in outcomes measured in adolescent major depressive disorder (MDD) RCTs has been described, the comprehensiveness of outcome reporting is unknown. This study aimed to assess the reporting of primary outcomes in RCTs evaluating treatments for adolescent MDD.

**Methods:**

RCTs evaluating treatment interventions in adolescents with a diagnosis of MDD published between 2008 and 2017 specifying a single primary outcome were eligible for outcome reporting assessment. Outcome reporting assessment was done independently in duplicate using a comprehensive checklist of 58 reporting items. Primary outcome information provided in each RCT publication was scored as “fully reported”, “partially reported”, or “not reported” for each checklist item, as applicable.

**Results:**

Eighteen of 42 identified articles were found to have a discernable single primary outcome and were included for outcome reporting assessment. Most trials (72%) did not fully report on over half of the 58 checklist items. Items describing masking of outcome assessors, timing and frequency of outcome assessment, and outcome analyses were fully reported in over 70% of trials. Items less frequently reported included outcome measurement instrument properties (ranging from 6 to 17%), justification of timing and frequency of outcome assessment (6%), and justification of criteria used for clinically significant differences (17%). The overall comprehensiveness of reporting appeared stable over time.

**Conclusions:**

Heterogeneous reporting exists in published adolescent MDD RCTs, with frequent omissions of key details about their primary outcomes. These omissions may impair interpretability, replicability, and synthesis of RCTs that inform clinical guidelines and decision-making in this field. Consensus on the minimal criteria for outcome reporting in adolescent MDD RCTs is needed.

## Background

Evidence-based health care is informed by randomized clinical trials (RCTs) and the synthesis of their results in meta-analyses and clinical practice guidelines. Inadequate reporting of clinical trials, including reporting of trial outcomes, remains a major challenge to evidence-based care [1, 2]. Outcomes are key trial components as they are the measured events that reflect the effect of an intervention on a participant (e.g., change in symptom severity, remission status) [3]. Empirical studies have shown that among the outcomes that are reported in trials, their descriptions often lack information regarding their selection, definition, measurement, and analysis [4–9]. Insufficiencies in the comprehensiveness of trial outcome reporting (i.e., the reporting provides enough detail to ensure full understanding of the outcome) not only limits the reproducibility and critical appraisal of findings, but also impedes research usability (e.g., translation to clinical practice) and contributes to a waste of research efforts and funding [1, 10]. While research examining outcome reporting has been performed in certain fields of pediatric health [7], to date, little attention to this issue has been seen in child and youth mental health.

Major depressive disorder (MDD) is a debilitating mental health condition that is currently one of the largest contributors of global disease burden [11]. MDD is prevalent among adolescent populations, affecting an estimated 5 % of youth aged 12 to 17 years [12, 13]. Adolescent MDD is associated with significant morbidity and mortality, including increased risk of suicide during adolescence and poor functional outcomes in adulthood [14–16]. Uncertainty remains when establishing evidence-informed effective treatments in this population, for example, whether medication adds to the effect of psychosocial therapy [17–20]. The uncertainty could be attributed, in part, to issues around the interpretability, replicability, and synthesis of RCT results that inform clinical guidelines and decision-making in this field. Variability in outcomes measured across RCTs is one contributing factor to these issues, such as interpretability of results, as reported in previous reviews [21, 22].

Recently, substantial heterogeneity was identified in the outcomes selected for measurement in clinical research studies in adolescent MDD, including RCTs [23, 24]. Also, given a single outcome, e.g., “depression symptoms”, up to 19 different outcome measurement instruments (OMIs) were used in current RCTs [23, 24]. Previous systematic reviews and meta-analyses of treatment interventions for adolescent depression suggest incomplete and variable reporting of outcome descriptions, such as a lack of information on how outcomes were measured [21, 25], but there has yet to be a systematic, transparent, and comprehensive thorough assessment of outcome reporting in this area.

Understanding the comprehensiveness of outcome reporting is important to assess whether this is a problem in this field and inform the potential need to standardize outcome reporting. To date, there has been no formal assessment of outcome reporting in published trial reports in adolescent MDD. The objective of this study was to evaluate whether outcome-specific information is reported in a comprehensive fashion in RCTs assessing depression treatments in adolescents.

## Methods

### Study selection

We performed our study in parallel with a systematic scoping review to identify eligible RCTs [24, 26]. RCTs that evaluated treatment for MDD in adolescents aged 12 to 18 years published in English between 2008 and 2017 were included; detailed search strategy and eligibility criteria are published elsewhere [27]. In brief, Medical Literature Analysis and Retrieval System Online (MEDLINE), Psychological Information Database (PsycINFO), and Cochrane Central Register of Controlled Trials (CCRCT) were searched to identify eligible RCTs. Title/abstract and full-text screening were performed independently and in duplicate.

For the purposes of this study, we employed additional eligibility criteria to restrict our sample to those RCTs that specified a single primary outcome. To identify trial reports with a single identifiable primary outcome, the following criteria were applied by the two reviewers, independently and in duplicate: (1) the outcome was explicitly specified as “primary” in the article text; (2) the outcome was explicitly described in the primary study objectives; and/or (3) the outcome was explicitly stated in the sample size calculation in the article text, as the primary outcome is used for sample size calculations [28]. We also included trials that reported on just one outcome, which we inferred to be the primary outcome.

### Outcome reporting assessment

To assess the comprehensiveness of outcome reporting, a candidate list of 70 outcome reporting items developed as part of the Instrument for reporting Planned Endpoints in Clinical Trials (InsPECT) project was used [29]. One aim of InsPECT was to develop a reporting extension to the Consolidated Standards of Reporting Trials (CONSORT) 2010 statement, which is an evidence-based, minimum set of recommendations for reporting randomized trials [30]. This new CONSORT extension, called CONSORT-Outcomes, is specific to outcomes and consists of a minimal, essential set of reporting items to be addressed for all primary and important secondary outcomes in any trial [29]. The final version of CONSORT-Outcomes was developed after the conduct of this study and is in preparation for peer-reviewed publication. The preliminary version of the extension used in this study is organized into 10 thematic categories: 1) What: Description of the outcome; 2) Why: Rationale for selecting the outcome; 3) How: The way the outcome is measured; 4) Who: Source of information of the outcome; 5) Where: Assessment location and setting of the outcome; 6) When: Timing of measurement of the outcome; 7) Outcome data management and analyses; 8) Missing outcome data; 9) Interpretation; and 10) Modifications [31–33].

Fifty-eight of the 70 candidate items were deemed to be relevant and/or possible to assess in this study. The 12 items excluded are described with reasons in Additional file 1 (e.g., the item “Specified if the outcome is part of a core outcome set, if a core outcome set is publicly available” was excluded because there is currently no core outcome set for adolescent depression).

Assessments of the comprehensiveness of outcome reporting in the published trials made using the resultant 58 item checklist were conducted independently and in duplicate by two reviewers (SP and AC) trained in clinical trial reporting methodology. A standardized data charting form (available online [34]) developed using Research Electronic Data Capture (REDCap) data management software [35] was used during assessment.

Outcome reporting was assessed and scored as “fully reported”, “partially reported”, or “not reported” for the primary outcome in each included RCT through the assessment of information reported for each of the 58 checklist items. An item was scored as “fully reported” when the entire concept of the item was reported in the published article. If the authors of the published RCT explicitly refer to supplementary materials (e.g., published protocols, statistical analysis plans, or secondary analysis reports) in relation to a specific reporting item, then this item would be scored as “fully reported” during assessment. A score of “partially reported” indicated that only one or some components of the item were reported; this applied only to checklist items that contained multiple components (see Table 2 for list of applicable items). For example, a score of “partially reported” for item #19 (validity of OMI in individuals similar to the study sample) indicates that the authors provided evidence of the validity of the OMI used, but did not specify whether the OMI was validated in individuals similar to the study sample. Items were classified as “not reported” when no information was provided for the item. Additional options included “unsure” and “not applicable”. “Unsure” was provided to flag content for discussion among reviewers to determine the appropriate reporting classification by consensus. A score of “not applicable” indicated that the item concept was not relevant to the RCT based on the information provided in the published article (e.g., the item “Specified whether order of administration of outcome measurement instrument was standardized, if assessing multiple outcomes” was only applicable to articles that reported using more than one outcome measurement instrument).

Prior to commencing the reporting assessment, the two reviewers (SP and AC) underwent a training procedure with an expert verifier (EJM), which was overseen by NJB, who led the development of the candidate reporting items as part of the InsPECT project [29]. To ensure sufficient agreement between the two reviewers, training was carried out on a random sample of three included RCTs [37–39]. The reviewers conducted outcome reporting assessment until sufficient inter-rater agreement was met for each of the three RCTs, which we defined as greater than 75% raw agreement. Initial percent agreement for the training set RCTs were 71, 77 and 89%. After discrepancies and areas of uncertainty were discussed, the two reviewers repeated their reporting assessment on the same sample of RCTs to ensure that there was a clear understanding of each checklist item concept. The percent agreement following discussion between the reviewers were 89, 96, and 97%, respectively. Remaining disagreements and areas of uncertainty were resolved by two expert team members (EJM and NJB). This same assessment and verification process were then applied to the remainder of the included RCTs such that agreement was reached on all items in the final data set.

### Synthesis of results

Data analysis included descriptive quantitative measures (counts and frequencies) of study characteristics and reporting item results. The comprehensiveness of reporting was calculated for each RCT based on the percentage of all items scored as “fully reported,” “partially reported,” and “not reported.”

## Results

### Search results

**Fig. 1:**
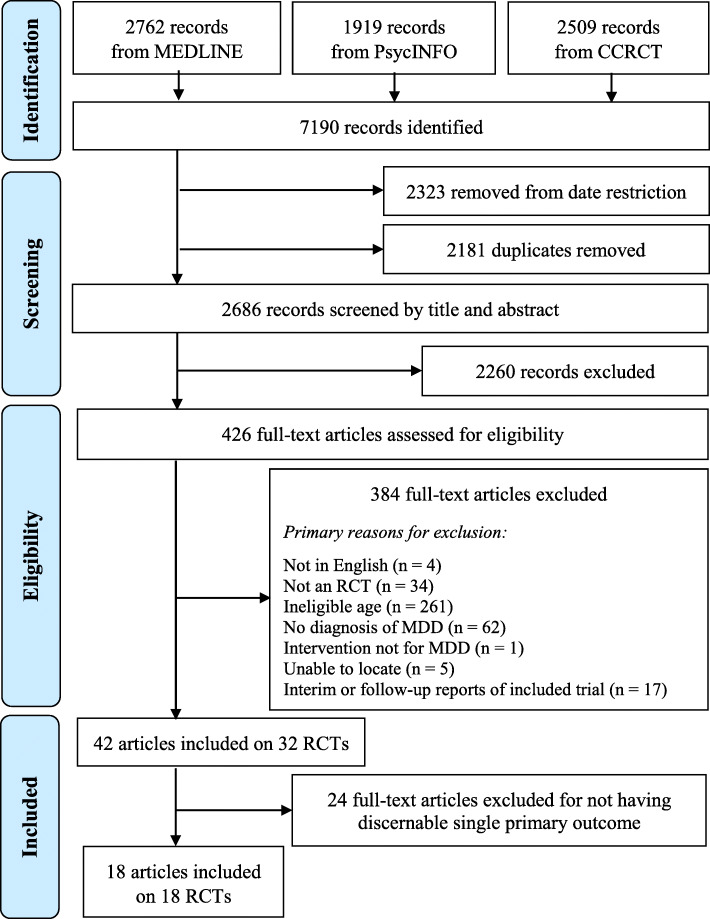
PRISMA flow diagram for trial reports assessing treatment interventions in adolescent major depressive disorder Included RCTs were from a systematic scoping review performed in parallel with our study [24]; eligibility criteria are published elsewhere [27]. RCT: randomized clinical trial; MDD: major depressive disorder.

Forty-two articles describing 32 RCTs were found after applying the initial eligibility criteria. Eighteen articles describing 18 unique RCTs were included in this study. Of these, 17 articles explicitly specified a single primary outcome and one article reported a single outcome, which was inferred to be the primary outcome. Twenty-four articles were excluded for not having a discernable single primary outcome. Figure 1 shows the flow diagram outlining this process.

### Study characteristics

Table 1 describes the 18 RCTs included in this study. The majority of included RCTs were carried out in North America (78%) and were government funded (61%). Nearly half of the RCTs examined drug therapy interventions (44%); however, other interventions included psychosocial therapy (39%), drug and psychosocial therapy (11%), and physical activity (6%). The median sample size was 188 (range: 22–470) study participants. Depressive symptom severity was the most commonly reported primary outcome, which was reported by 12 of the 18 included RCTs.

**Table 1:** Characteristics of included articles of randomized clinical trials assessing treatment interventions for adolescents with major depressive disorder (*n* = 18 trials)

First author, year of publication [citation]	Intervention type^a^	Age of included population (years)	Total sample size at enrolment	Length of follow-up (weeks)	Study site location	Funding source^b^	Primary outcome
Cheung et al., 2008 [40]	Drug	13–19	22	52	North America	Government	Time to relapse
Goodyer et al., 2008 [41]	Drug and Psychosocial	11–17	208	28	Europe	Government	Global measure of current mental health status
Emslie et al., 2009 [42]	Drug	12–17	316	8	North America	Industry	Depressive symptom severity
Findling et al., 2009 [43]	Drug	12–17	34	8	North America	Not for Profit; Industry	Depressive symptom severity
Jacobs et al., 2010 [44]	Drug and Psychosocial	12–17	439	12	North America	Government	Oppositionality
Atkinson et al., 2014 [39]	Drug	7–17	337	36	North America	Industry	Depressive symptom severity
DelBello et al., 2014 [45]	Drug	12–17	308	12	North America	Industry	Depressive symptom severity
Emslie et al., 2014 [46]	Drug	7–17	448	36	North America	Industry	Depressive symptom severity
Richardson et al., 2014 [47]	Psychosocial	13–17	101	52	North America	Government	Depressive symptom severity
Szigethy et al., 2014 [37]	Psychosocial	9–17	217	12	North America	Government	Depressive symptom severity
Clarke et al., 2016 [38]	Psychosocial	12–20	41	26	North America	Government	Depression remission
Kobak et al., 2015 [48]	Psychosocial	12–17	65	12	North America	Government	Depressive symptom severity
Charkhandeh et al., 2016 [49]	Psychosocial	12–17	188	12	Asia	N/R^c^	Depressive symptom severity
Cheung et al., 2016 [50]	Drug	13–18	25	24	North America	Government	Time to relapse
Kondo et al., 2016 [51]	Drug	13–20	33	8	North America	Government	Frontal lobe phosphocreatine levels
McCauley et al., 2016 [52]	Psychosocial	12–18	60	52	North America	Government; Hospital	Depressive symptom severity
Goodyer et al., 2017 [17]	Psychosocial	11–17	470	86	Europe	Government	Depressive symptom severity
Wunram et al., 2017 [53]	Physical Activity	13–18	64	26	Europe	Not for profit; Hospital	Depressive symptom severity

^a^Intervention types categorized as follows: Drug (i.e., duloxetine, fluoxetine, citalopram, sertraline, escitalopram); Psychosocial (i.e., cognitive behavioural therapy, collaborative care intervention, supportive non-directive therapy, Reiki method); Physical activity (i.e., whole-body vibration, cardiovascular training)

^b^Funding sources categorized as follows: Government: funded by a governmental organization (i.e., Canadian Institutes of Health Research, National Institute of Mental Health, National Institute for Health Research); Hospital: funded by hospital or university-affiliated research centre within hospital (i.e., University of Washington/Seattle Children’s Hospital Institute of Translational Health Sciences Pediatric Clinical Research Center); Industry: for profit corporation (i.e., Eli Lilly and Company, Somerset Pharmaceuticals Incorporated, Forest Laboratories); Not for profit: not for profit foundation or organization (i.e., Marga and Walter Boll Foundation, American Foundation for Suicide Prevention, St. Luke’s Foundation of Cleveland)

^c^*N/R*: not reported

### Outcome reporting assessment

**Fig. 2:**
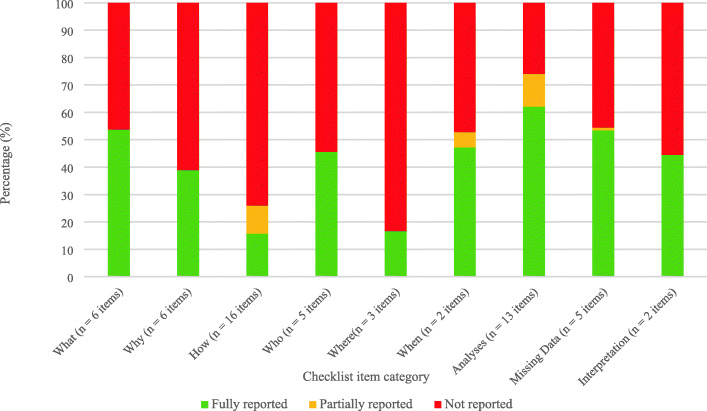
Outcome reporting comprehensiveness across 18 adolescent major depressive disorder trials, by thematic item category

The overall percentage of items scored as “fully reported”, “partially reported”, or “not reported” was variable across the thematic categories (Fig. 2). The highest percentage of fully reported items was “Outcome data management and analyses” (62%), followed by “Missing outcome data” (54%) and “What: Description of the outcome” (53%). The categories of “How: The way the outcome is measured” and “Where: Assessment location and setting of the outcome” had the lowest percentage of “fully reported” items (16 and 17%, respectively).

**Fig. 3:**
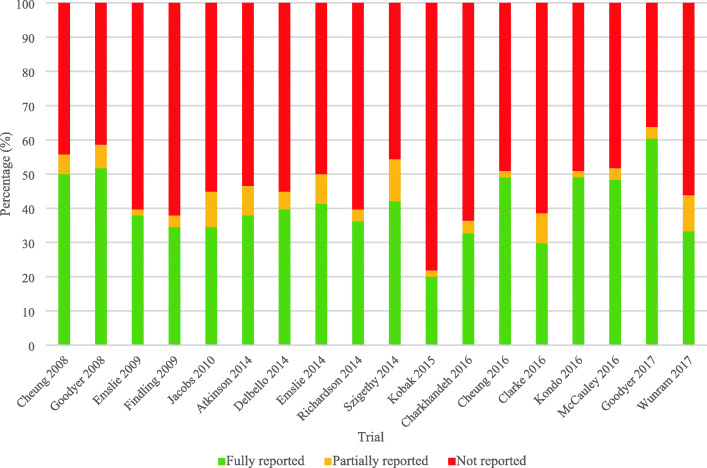
Outcome reporting comprehensiveness across 18 adolescent major depressive disorder trials

There was wide variability in how each of the 18 included RCTs scored on the assessments of outcome comprehensiveness; reporting frequencies for all 58 items are presented in Table 2. Overall, about half of the 58 items were fully reported per RCT (Fig. 3; Additional file 2). The median percentage of items that were scored as fully reported was 38% (range: 20–60%) per RCT (Fig. 3). The percentage of reporting items fully reported appeared relatively stable over the 10 year time period (2008 to 2017) (Fig. 3). Notable findings for items in each outcome reporting category are described below.

**Table 2:** Frequency of outcome reporting classifications for each reporting itemfor the primary outcome in 18 included RCTs

Reporting Item Thematic Item Category and Number	Fully reported N (%)	Partially reported N (%)^a^	Not reported N (%)
What: Description of the outcome			
1. Described the outcome domain^b^	6 (33)	N/A	12 (67)
2. Stated the outcome	18 (100)	N/A	0 (0)
3. Specified the outcome as primary	17 (94)	N/A	1 (6)
4. Provided a rationale for classifying the outcome as primary, instead of secondary	3 (17)	N/A	15 (83)
5. Defined clinical significance on the outcome (e.g., minimal important difference, responder definition), including what would constitute a good or poor outcome	11 (61)	N/A	7 (39)
6. Justified the criteria used for defining meaningful change (e.g., the minimal important difference, responder definition), including what would constitute a good or poor outcome, such as from an outcome measurement interpretation guideline	3 (17)	N/A	15 (83)
Why: Rationale for selecting the outcome			
7. Explained how the outcome addresses/relates to the hypothesis of the study	8 (44)	N/A	10 (56)
8. Explained how the outcome addresses the objective/research question of the study (i.e., to compare the effect of intervention A versus intervention B on outcome X)	10 (56)	N/A	8 (44)
9. Described why the outcome is relevant to each stakeholder group involved in this trial (e.g., patients, decision makers, policy makers, clinicians, funders, etc.)	11 (61)	N/A	7 (39)
10. Reported which stakeholders (e.g., patients, decision makers, policy makers, clinicians, funders, etc.) were actively involved in outcome selection	2 (11)	N/A	16 (89)
11. Explained the mechanism (e.g., pathophysiological, pharmacological, etc.) or theoretical framework/model by which the experimental intervention is expected to cause change in the outcome in the target population	7 (39)	N/A	11 (61)
12. Provided rationale for the choice of the specific type of outcome (e.g.,., why a patient-reported outcome instead of a clinician reported outcome)	4 (22)	N/A	14 (78)
How: The way the outcome is measured			
13. Described the outcome measurement instrument used. This should include instrument scaling and scoring details (e.g., range and direction of scores)^c^	9 (50)	8 (44)	1 (6)
14. Specified whether the outcome measurement instrument will be used in accordance with any user manual and specify and justify deviations if planned	0 (0)^d^	N/A	16 (100)
15. Specified a recall period for outcome measurement instrument	1 (6)	N/A	17 (94)
16. Described mode of outcome assessment (e.g., face to face, telephone, electronically)	9 (53)^d^	N/A	8 (47)
17. Justified the mode of outcome assessment (e.g., justification for equivalence between different modes of administration, if applicable)	1 (6)^d^	N/A	16 (94)
18. Described any additional resources/materials or processes when performing outcome assessment, when relevant (e.g.,, a stethoscope, language interpreter, fasting prior to colonoscopy, etc.)	5 (28)	N/A	13 (72)
19. Described or provided reference to an empirical study that established validity of the outcome measurement instrument in individuals similar to the study sample (i.e., measures what it is supposed to measure)^c^	1 (6)^d^	3 (19)	12 (75)
20. Described or provided reference to an empirical study that established validity of the outcome measurement instrument in the study setting (i.e., measures what it is supposed to measure)^c^	1 (6)^d^	3 (19)	12 (75)
21. Specified whether more than one language version of the outcome measurement instrument was used and state whether translated versions have been developed using currently recommended methods, if applicable	0 (0)	0 (0)	15 (83)^d^
22. Described or provided reference to an empirical study that established reliability of the outcome measurement instrument in individuals similar to the study sample (i.e., ability to produce consistent results)^c^	3 (17)	6 (33)	9 (50)
23. Described or provided reference to an empirical study that established reliability of the outcome measurement instrument in the study setting (i.e., ability to produce consistent results)^c^	0 (0)	7 (39)	11 (61)
24. Described or provided reference to an empirical study that established the responsiveness of the outcome measurement instrument in the study sample (i.e., ability to detect change over time given a change in disease activity or status)	2 (13)^d^	0 (0)	14 (87)
25. Described the feasibility of the outcome measurement instrument in the study sample (i.e., the practical considerations of using an instrument, including its ease of use, time to complete, monetary costs, and interpretability of the question(s) included in the instrument)	5 (28)	0 (0)	13 (72)
26. Described the acceptability and burden of the outcome measurement instrument in the study sample^c^	2 (11)	1 (6)	15 (83)
27. Specified whether order of administration of outcome measurement instrument was standardized, if assessing multiple outcomes	2 (12)^d^	N/A	15 (88)
28. Specified whether outcome data will be monitored during the study to inform the clinical care of individual trial participants, and if so, how this will be managed in a standardized way	2 (11)	0 (0)	16 (89)
Who: Source of information of the outcome			
29. Described who (e.g., nurse, occupational therapist, technician, parent, outcome adjudicators), and if applicable, how many, assessed the outcome in each study group	9 (50)	0 (0)	9 (50)
30. Justified the choice of outcome assessor(s) (e.g., proxy versus healthcare provider)	3 (17)	N/A	15 (83)
31. Described whether the outcome assessor(s) were blinded/masked to intervention assignment	14 (78)	N/A	4 (22)
32. Described any study-specific training required for outcome assessors to apply the outcome measurement instrument	6 (33)	N/A	12 (67)
33. Described how outcome data quality was maximized (e.g., duplicate measurements)	9 (50)	N/A	9 (50)
Where: Assessment location and setting of the outcome			
34. Specified geographic location of outcome assessment for each study group (e.g., list of countries where outcome data was collected)	5 (28)	N/A	13 (72)
35. Described setting of outcome assessment for each study group (e.g., clinic, home, other)	4 (22)	N/A	14 (78)
36. Justified suitability of the outcome assessment setting(s) for the study sample (e.g., family doctor office vs. home when measuring blood pressure)	0 (0)	N/A	18 (100)
When: Timing of measurement of the outcome			
37. Specified timing and frequency of outcome assessment(s) for outcomes (e.g., time point for each outcome, time schedule of assessments)^c^	16 (89)	2 (11)	0 (0)
38. Provided justification of timing and frequency of outcome assessment(s) (such as pathophysiological or epidemiological evidence for disease processes and complications to occur and/or pragmatic justification)	1 (6)	0 (0)	17 (94)
Outcome data management and analyses			
39. Provided definition of outcome analysis population	16 (89)	N/A	2 (11)
40. Described unit of analysis of the outcome (i.e., cluster or individual)	18 (100)	N/A	0 (0)
41. Described outcome analysis metric (e.g., change from baseline, final value, time to event)	18 (100)	N/A	0 (0)
42. Described method of aggregation for outcome data (e.g., mean, median, proportion)	18 (100)	N/A	0 (0)
43. Described statistical methods and/or significance test(s) (name or type) used for analyzing outcome data. This should include any analyses undertaken to address multiplicity/type I (α) error, particularly for trials with multiple domains and time points^c^	11 (61)	7 (39)	0 (0)
44. Described the covariates and factors in the statistical model (e.g., adjusted analyses) used for analyzing outcome data, if applicable	14 (82)^d^	N/A	3 (18)
45. Provided justification for covariates and factors and why they were selected, if applicable	2 (12)^d^	N/A	15 (88)
46. Described results for each group, including estimated effect size and its precision (such as 95% confidence interval). For binary outcomes, presentation of both absolute and relative effect sizes is recommended^c^	14 (78)	4 (22)	0 (0)
47. Described time period (i.e., chronological time since randomization) for which the outcome was analyzed	18 (100)	N/A	0 (0)
48. Described outcome data, assessment process, and analysis for participants who discontinued or deviated from the assigned intervention protocol^c^	1 (6)	15 (83)	2 (11)
49. Described outcome data entry, coding, security and storage, including any related processes to promote outcome data quality (e.g., double entry, range checks from outcome data values)	0 (0)	1 (6)	17 (94)
50. Described blinding procedure(s) applied to data entry personnel and/or data analysts	3 (17)	0 (0)	15 (83)
51. Described methods for additional analyses (e.g., subgroup analyses), if applicable	8 (62)^d^	N/A	5 (38)
Missing outcome data			
52. Described how much outcome data was missing	16 (89)	N/A	2 (11)
53. Described any reasons for missing outcome data in each arm (e.g.,, reasons for withdrawal or reasons for lack of follow-up), with enough detail that the reported reason can be used to reduce the uncertainty about the potential underlying mechanism of missing outcome data	11 (65)^d^	0 (0)	6 (35)
54. Explained statistical methods to handle missing outcome items or entire assessments (e.g., multiple imputation)	13 (72)	N/A	5 (28)
55. Provided justification for methods used to handle missing outcome data. This should include: (1) assumptions underlying the missing outcome data mechanism with justification (including analyses performed to support assumptions about the missingness mechanism); and (2) how the assumed missingness mechanism and any relevant features of the outcome data would influence the choice of statistical method(s) to handle missing outcome data including sensitivity analyses^c^	3 (17)	1 (6)	14 (77)
56. Described any outcome analyses conducted to assess the risk of bias posed by missing outcome data (e.g., comparison of baseline characteristics of participants with and without missing outcome data)	3 (18)^d^	N/A	14 (82)
Interpretation			
57. Interpreted outcome data in relation to clinical outcomes, where relevant	12 (67)	N/A	6 (33)
58. Discussed impact of missing outcome data on the interpretation of findings, if applicable	4 (22)	N/A	14 (78)

^a^*N/A* not applicable, refers to instances where “partially reported” was not a valid scoring option. Items scored as “Not applicable” were excluded from the overall assessment calculations because they were deemed not relevant to the assessment of outcome reporting by the research team (AM, EM, SP, NJB) by consensus

^b^Outcome domain defined as: “A relatively broad aspect of the effect of illness on a child, within which an improvement may occur in response to an intervention. In general these domains may not be directly measurable themselves, so outcomes are selected to assess change within them.” [36]

^c^Item was considered “fully reported” only when all components (e.g., timing AND frequency, item 37) within each checklist item were “fully reported”

^d^Several items do not total to adenominator of N = 18 trials, for the following reasons: Items 16 and 17 (denominator = 17): item was not applied to articles where the primary outcome was a biomarker. Items 14, 19–20, 24 (denominator = 16): item was not applied to articles where the outcome was assessed using clinician judgement. Item 21 (denominator = 15): item not applied to articles with biomarkers or time to event as the primary outcome. Item 27 (denominator = 17): item was not applied to articles where only one outcome measurement instrument was reported. Item 44 and 45 (denominator = 17): items were only applicable to articles with statistical methods that include covariates/factors. Item 51 (denominator = 13): item was only applied to articles that included additional/subgroup analyses. Item 53 and 56 (denominator = 17): items were only applied to articles that reported having missing data

#### What: description of the outcome

All 18 RCTs explicitly stated the outcome and 94% specified the outcome as primary explicitly in the article text (items #2 and #3, respectively). Criteria for clinical significance on the primary outcome was defined in 61% of RCTs (item #5). Items that were less frequently reported in this category were the description of the outcome domain (definition provided in Table 2; 33%; item #1), the justification for the selection of the primary outcome (17%; item #4), and justification of the criteria used to define clinical significance (17%; item #6).

#### Why: rationale for selecting the outcome

Items describing the rationale for selecting the outcome were variably reported. The most frequently reported items included describing why the primary outcome is relevant to stakeholders (61%; item #9) and describing how the primary outcome addresses the objective/research question of the study (56%; item #8). The least frequently reported item involved describing which stakeholders were actively involved in the selection of the primary outcome (11%; item #10).

#### How: the way the outcome is measured

Most RCTs described the OMI used (94%; item #13), but less than half included details about the scoring and scaling of the OMI (44%; item #13). Few RCTs described the measurement properties of the OMI. Only 16 of the 18 RCTs could be assessed for reporting on the validity of the OMI as two RCTs used clinician judgment. One of the 16 RCTs described both the validity of the OMI in individuals similar to the study sample (item #19) and in the study setting (item #20). Three of the 16 RCTs reported on the validity of the OMI without specifying if the validity was established in similar study samples or settings (19%; items #19 and #20). Three RCTs reported on the reliability of the OMI in a relevant study sample (item #22), and none reported on the reliability of the OMI specific to the study setting (item #23). Six of the 16 RCTs reported the reliability of the OMI but did not specify whether reliability was established in individuals similar to the study sample and seven studies did not specify reliability in the setting used. Few RCTs explicitly specified responsiveness of the OMI used (13%; item #24) or the feasibility (28%; item #25), acceptability and/or research burden (11%; item #26) of the OMI in the study sample.

#### Who: source of information of the outcome

Details about who the outcome assessors were, and the number of assessors, were fully reported in half of the included 18 RCTs (item #29), but justification as to who the assessors were (e.g., trained clinicians, a nurse, or parents) was lower (17%; item #30). The masking (blinding) status of outcome assessors to intervention assignment was frequently fully reported (78%; item #31). Training of assessors and a description of how outcome data quality was maximized were reported in over 33 and 50% of RCTs, respectively (items #32 and #33).

#### Where: assessment location and setting of the outcome

The location and setting of outcome assessment were not frequently reported in the included RCTs (28 and 22%; items #34 and #35, respectively). No RCT provided justification of the suitability of the outcome assessment setting for the study sample (item #36).

#### When: timing of measurement of the outcome

Timing and/or frequency of outcome assessment were reported in all RCTs (item #37), though justification was provided in just 6% of RCTs (item #38).

#### Outcome data management and analyses

Overall, items describing outcome data management and analyses details were well reported. All RCTs described the unit of analysis of the outcome (item #40), the outcome analysis metric (item #41), the method of aggregation (item #42), and the time period for which the outcome was analyzed (item #47). Items that were less frequently reported included the justification for the selection of covariates/factors used for analyzing outcome data (12%; item #45) and the precision of the estimated effect size as part of the outcome results (22%; item #46).

Details about outcome data management were variably reported, ranging from 0 to 17% of items being fully reported (items #48–50). Most RCTs described some information pertaining to the outcome data, assessment process, and/or analysis for participants who discontinued or deviated from intervention protocol (83%; item #48).

#### Missing outcome data and interpretation

Over 60% of the RCTs described how much outcome data were missing, reasons for missing outcome data, and the statistical methods to handle missing outcome data (items #52–54). Justification for the methods used to handle missing outcome data was the least frequently reported item in this category (17%; item #55). Most RCTs provided an interpretation of outcome data in relation to clinical outcomes (67%; item #57). Few discussed the potential impact of missing outcome data on the interpretation of findings (22%; item 58).

## Discussion

To the best of our knowledge, this is the first study to conduct an in-depth assessment of the comprehensiveness (i.e., reporting that provides enough detail to ensure full understanding of an outcome) of the descriptions of primary outcomes in publications of adolescent MDD RCTs. We found that nearly 60% of articles did not report a discernable single primary outcome. Primary outcome descriptions varied considerably in the level of detail provided between trial publications and by the type of information. On average, approximately half of the reporting items from the 58 item checklist were not fully reported in the published articles. Notably, the overall comprehensiveness of reporting has been relatively stable over the 10-year period, with no notable improvement. Items describing the analysis of the primary outcome were frequently reported whereas items describing how the primary outcome was measured and where assessments took place were reported in less than 20% of RCTs.

Comprehensive outcome reporting enables transparency and reproducibility of information about the planning, conduct, and findings of trials [54]. Consequently, knowledge users are able to critically evaluate and utilize trial results to inform effective clinical practice and decision-making at the health system level. Conversely, when trial outcome descriptions are insufficiently or variably reported, this can impair the use of trial results in evidence syntheses (i.e., meta-analyses) and effectively reduce the ability to interpret these results for clinical decision-making [10]. In the text that follows, we discuss potential reasons for the current state of primary outcome reporting in publications of adolescent depression RCTs and examine implications for the interpretability, replicability, and synthesis of such RCTs that inform clinical guidelines and decision-making in the field.

### Outcome reporting

It is important to note that while there was variation in primary outcome reporting across the included RCTs, there were also aspects that were consistently well reported. These included details about the timing and frequency of outcome assessment, as well as masking status of outcome assessors. Additionally, details about primary outcome analyses were generally well reported. All RCT publications described the unit of analysis, the method of aggregation, the analysis metric, and the time period for which the outcome was analyzed. This may be attributed to the fact that items on timing of outcome assessment, masking of outcome assessors, and outcome analysis are iterations of existing reporting items from the CONSORT statement [30]. First published in 2001, CONSORT is a widespread and highly endorsed reporting guideline that has previously been shown to be beneficial to the comprehensiveness of trial reporting in published articles [55]. Though CONSORT provides general guidance on how to report outcomes, deficiencies in outcome reporting in trial publications remains, as demonstrated in our study and in previous publications on outcome reporting [8, 9, 56–58]. The uptake of the CONSORT-Outcomes standard may help ensure that all essential outcome-specific information is reported in future youth depression and other trial publications [29].

Though most details about primary outcome analyses were frequently fully reported, reporting deficiencies were found particularly for details on covariate selection for adjusted analyses. Out of the 17 RCTs that reported adjusted analyses among the 18 included RCTs, two provided a justification for the selection of the included covariates. For example, while the authors of one trial wrote that covariates were included “... to model systematic differences due to treatment, assessment time point or patient characteristics,” no explanation was provided as to why the latter two covariates were selected [17]. Previous reviews report similar deficiencies in the justification of adjusted analyses [59–61]. A lack of explanation for the covariates used can obscure the presence or absence of “data torturing” in the reported analyses [62], where the covariates in the adjusted outcome analysis are selectively chosen post-hoc to demonstrate or exaggerate positive treatment effects. This inflates type I error rates and can impact meta-analyses findings when inflated effect estimates are pooled [63].

Our study showed that details on the description of the OMI used, including its measurement properties (e.g., validity, reliability, responsiveness), are not frequently reported in a comprehensive manner. The importance of reporting this information is illustrated by a recent review, which found that providing a description of the trial OMI(s), including details on their measurement properties, is one of the most consistently recommended pieces of information to include in clinical trial reports among documents that provide guidance on outcome reporting in trials [64]. Reporting this information is essential to communicate the validity of outcome results.

### Implications for patients, caregivers, and healthcare providers

There are wide implications of these findings for stakeholders of mental health trials, such as patients, caregivers, and healthcare providers. For example, the item “Justified the criteria used for defining meaningful change (e.g., the minimal important difference, responder definition) including what would constitute a good or poor outcome” can provide useful context and guidance for clinical practice when fully reported. However, only 17% of RCTs included in our study provided a justification of the criteria used to defining meaningful change. Given the growing movement of patient and caregiver engagement in research, care providers may expect justification for these criteria that they can evaluate and include in consultations with patients and caregivers when discussing possible treatment outcomes. This expectation is supported by a review by Beaton et al that emphasized the importance of different perspectives when defining minimal clinically important differences (e.g., meaningful change) [65]. This deficiency of reporting suggests that engagement from important stakeholders remains uncommon in determining what constitutes meaningful change. More typically, the determination of a good or poor outcome is based on statistical interpretations rather than what meaningful change means from a patient, caregiver, or healthcare provider perspective [66, 67].

Another outcome-specific reporting item that plays a role in clinical decision-making in youth mental health is the justification of the timing and frequency of outcome assessment. Major depressive episodes often improve and remit within seven to nine months of symptom onset, when untreated, and the time to symptom improvement and remission rates vary depending on the type of treatment [68, 69]. Information on expected time to symptom improvement and time to remission is an imperative consideration when deciding on the timing and frequency of outcome assessment for RCTs in adolescent MDD, as change should be measured prior to natural remission, or in line with the mechanism of action of the therapy provided, in order to detect change due to treatment intervention [70]. As only one RCT included in our study justified the choice of time points and schedule of outcome assessment, this can leave knowledge users with little evidence to appropriately interpret the trial results.

Variation in outcome reporting can place a hindrance on the usability and comprehension of trial findings by knowledge users, who often include those deciding on the best course of care for their patients, other researchers, and importantly, also patients and caregivers themselves. Clear reporting of the rationale for selecting the primary outcome in a trial, for example, would help improve patient understanding about the primary trial objectives and its relevance to the domain in which they seek to see improvement themselves (i.e., whether an outcome that is meaningful to them was measured). However, this information was reported in only three of 18 RCTs included in our study. Relatedly, only a small minority of articles described stakeholder involvement in outcome selection. As yet, there has been no assessment involving key stakeholders about which outcomes should be selected and measured in adolescent MDD trials [71, 72]. Outcomes can be selected for use in a trial for a variety of reasons (e.g., clinical importance, patient acceptability, cost, historical reasons), with different importance placed on different outcomes by different stakeholders [70, 73]. The development of a core outcome set (COS), an agreed minimum set of outcomes that should be measured [74], could help with standardization of outcome selection and reporting in adolescent MDD trials, especially when coupled with reporting guidance, such as CONSORT-Outcomes. Although COS is still a relatively new concept for use in mental health trials [71], the Core Outcome Measures in Effectiveness Trials (COMET) Initiative has fostered the development of COS in many other clinical areas [75]. The development of a COS for adolescent MDD is underway [27, 76].

It is important to note, however, that journal word limits may preclude researchers from reporting all these outcome-specific details in their final trial report, and not all may be relevant to all trials. In cases where these limits are in place, online supplementary material or appendices provided by journals, online open source repositories, and other study documents such as publicly available trial protocols, statistical analysis plans, and clinical study reports may serve to supplement any important details excluded from a trial report. The use of these additional materials can be advantageous for clinical researchers to optimize the transparent reporting of trial objectives, methods, and results that are interpretable and useful for knowledge end-users.

### Strengths and limitations

We conducted a comprehensive assessment of primary outcomes in RCTs spanning a range of reporting categories. These categories originated from preliminary results of a scoping review of the literature and consultation with mental health content experts and methodologists [64]. We focused on primary outcomes in this study given they are the most important outcomes in a trial as they drive primary study objectives, sample size calculations, and are key in the interpretation of trials’ identified treatment effect sizes [28]. We employed rigorous data capture methods for our study by having two trained reviewers with experience in epidemiologic methods and clinical research perform reporting assessments in duplicate and using a consensus-based approach to resolve any discrepancies in scoring classifications.

One limitation of this study may be that we excluded publications of RCTs with multiple primary outcomes and RCTs that were unclear in identifying their primary outcomes. Therefore, the actual state of primary outcome reporting in adolescent MDD trials could potentially differ from what we found in our study. Notably, previous reviews examining outcome reporting in published mental health trials found reporting deficiencies in the specification of which outcomes were primary or secondary and evidence of selective outcome reporting of multiple primary outcomes [5, 77]. Second, we excluded grey literature as an information source and restricted to adolescent MDD trials published in English, thereby potentially reducing the generalizability of our findings. Nevertheless, we suspect that outcome reporting is also variable in RCTs not published in English, based on similar findings from a review with no language restrictions that assessed reporting of RCTs in CONSORT-endorsed journals [55]. Third, there may be some subjectivity in our distinction between “fully reported” and “partially reported” for items where both reporting scores were applicable. To help mitigate this risk, two trained reviewers performed the assessment in duplicate accompanied by a training guide – developed by the research team (AM, EJM, SP, AC) with expertise from methodology experts (NJB, MO) – that explained each reporting item and scoring category with descriptive examples. Finally, the assessment tool used in our study is an early version of a new CONSORT-Outcomes extension and has not yet been validated. As this study was conducted while CONSORT-Outcomes was in early development, we included all 58 items in our study knowing that some items may be more relevant to trials in adolescent depression than others. Examples of more relevant items may include justifying the timing and frequency of outcome assessment and reporting which stakeholders were actively involved in selecting the primary outcome.

## Conclusions

Large heterogeneity exists in primary outcome reporting in published RCTs in adolescent MDD, with frequent omissions of key details about outcome selection and measurement. These omissions may impair interpretability, replicability, and synthesis of RCTs that inform clinical guidelines and decision-making in this field. A standard with minimal criteria for outcome reporting in RCTs in adolescent MDD RCTs is needed.

## Supplementary information

**Additional file 1: Table S1.** Outcome reporting items removed from the comprehensive item checklist. Word document.

**Additional file 2.** Outcome reporting comprehensiveness across 18 adolescent major depressive disorder trials, by checklist item. Excel document.

## Data Availability

The datasets generated and/or analyzed during the current study are available in the Open Science Framework repository, https://osf.io/teqap/.

## Abbreviations

RCT: Randomized clinical trial
MDD: Major depressive disorder
OMI: Outcome measurement instrument
MEDLINE: Medical Literature Analysis and Retrieval System Online
PsycINFO: Psychological Information Database
CCRCT: Cochrane Central Register of Controlled Trials
InsPECT: Instrument for reporting Planned Endpoints in Clinical Trials
CONSORT: Consolidated Standards of Reporting Trials
REDCap: Research Electronic Data Capture
COS: Core Outcome Set
COMET: Core Outcome Measures in Effectiveness Trials
