# Supplementary material for: Primary outcome reporting in adolescent depression clinical trials needs standardization
Source: BMC Med Res Methodol. 2020 May 25;20:129. doi: 10.1186/s12874-020-01019-6 (PMC7247139; doi:10.1186/s12874-020-01019-6)
Supplement: Supplementary file 1 — Additional file 1: Table S1. Outcome reporting items removed from the comprehensive item checklist. Word document. [file 12874_2020_1019_MOESM1_ESM.docx]

Supplementary Table 1. Outcome reporting items removed from the comprehensive item checklist^a^

| Outcome reporting item | Reason for exclusion |
| --- | --- |
| What: Description of the outcome |  |
| If reporting a composite outcome (i.e., two or more component outcomes that are combined), all individual components are defined. | There were no composite outcomes in the sample of included studies, so this item did not apply. |
| If there are other published definitions of the outcome, explained why the chosen definition was used. | There was no indication in the included studies that other published definitions of the outcome exist so this item did not apply. |
| Why: Rationale for selecting the outcome |  |
| Specified if the outcome is part of a core outcome set, if a core outcome set is publicly available. If so, refer to which core outcome set it is part of (e.g., via www.comet-initative.org/). | There is currently no core outcome set for adolescent depression, so this item did not apply. |
| If completely new outcome, justified why other outcomes are not appropriate or relevant. | There was no indication in the included studies that the reported primary outcome is completely new so this item did not apply. |
| Who: Source of information of the outcome |  |
| Specified the name, affiliation, and contact details for the individual(s) responsible for the outcome content to identify the appropriate point of contact for resolution of any outcome-specific inquiries. | This item concept is considered a trial-level reporting item and not an outcome-level reporting item, therefore it is out of scope for assessing the outcome reporting of the included trial reports. |
| Outcome data management and analyses |  |
| Provided the results of all planned outcome analyses that were undertaken, regardless of statistical significance. | This item requires access to the protocols or statistical analysis plans of the included studies (if publicly available) which was not done in this study. |
| Described how unplanned repeat measurements were handled when analyzing outcome data (e.g., repeat blood pressure result in patient due to initial abnormal reading). | There was no indication in the included studies that unplanned repeat measurements occurred, so this item did not apply. |
| If someone other than a member in the study group analyzed the outcome data, described the person’s affiliations (e.g., if the person is affiliated with industry) | There was no indication in the majority of included studies that someone outside the study group analyzed the outcome data, so this item could not be assessed. |
| Describe any plans to minimize missing outcome data | This item concept is out of scope for assessing the outcome reporting of the included trial reports (deemed more applicable to outcome reporting in trial protocols). |
| Interpretation |  |
| Described other considerations or procedures that could affect the ability to interpret the outcome results (e.g., for per protocol analysis, describe the limitations in the methods used to monitor subject compliance) | The item concept is subjective in nature and could not be assessed in a standardized way in the majority of included studies. |
| Modifications | |
| Described any changes to trial outcomes after the trial commenced, with reasons | There was no indication in the majority of included studies that changes to trial outcomes occurred, so this item could not be assessed. |
| Described if there were any changes made to the planned analysis of outcomes (including omissions) after the trial commenced, and if yes, provided justification. For example, if any pre-specified covariates are omitted in the model, describe which co-variates were omitted and justify the omissions, including any statistical methods employed to justify this omission | There was no indication in the majority of included studies that changes to planned analysis of outcomes occurred, so this item could not be assessed. |

^a^Full checklist version available at <https://osf.io/8nejr/>.
